# A Hidden Diagnosis Behind Giant Inverted T-waves: Recognizing Apical Hypertrophic Cardiomyopathy in a Young Adult

**DOI:** 10.7759/cureus.91364

**Published:** 2025-08-31

**Authors:** Abdul Rehman, Muzamil Aslam Chaudhary, Muhammad Yahya Khan, Syeda Zoya Chishti, Fazeelah Bibi

**Affiliations:** 1 Emergency Department, St. Luke's General Hospital, Kilkenny, IRL; 2 Emergency Department, Wrightington, Wigan and Leigh NHS Foundation Trust, Wigan, GBR; 3 General Surgery Department, Allied Hospital Faisalabad, Faisalabad, PAK; 4 Pediatric Surgery Department, Children Hospital Faisalabad, Faisalabad, PAK; 5 Obstetrics and Gynaecology Department, Pakistan Institute of Medical Sciences Hospital, Islamabad, PAK; 6 Emergency Department, Pakistan Institute of Medical Sciences Hospital, Islamabad, PAK

**Keywords:** acute coronary syndrome, apical hypertrophic cardiomyopathy, cardiac mri, chest pain, yamaguchi syndrome

## Abstract

Chest pain in young adults can pose a diagnostic challenge, often raising concerns for acute coronary syndrome but more commonly resulting from non-cardiac causes. We report a case of apical hypertrophic cardiomyopathy (ApHCM) in a 27-year-old man, presenting with episodic chest pain and striking ECG abnormalities but normal coronary angiography. This case underscores the value of recognizing characteristic ECG patterns and the role of cardiac MRI in confirming ApHCM, a rare but clinically significant condition that can otherwise be misdiagnosed. Awareness of these findings in young patients without traditional cardiovascular risk factors is essential, as early MRI can establish the diagnosis and prevent unnecessary invasive procedures.

## Introduction

Chest pain in young individuals is frequently non-cardiac in origin, but it can occasionally reveal rare cardiac conditions. Hypertrophic cardiomyopathy (HCM) is a genetically heterogeneous myocardial disease characterized by unexplained left ventricular hypertrophy in the absence of secondary causes. Apical hypertrophic cardiomyopathy (ApHCM), a rarer form first described in Japan, is typically seen in middle-aged men of East Asian descent. It is distinguished by hypertrophy localized predominantly to the left ventricular apex. While often considered a benign variant, ApHCM may mimic acute coronary syndromes (ACS), especially when presenting with chest pain and ischemic-appearing electrocardiogram (ECG) changes [1].

The present case highlights the diagnostic challenge posed by ApHCM when presenting outside its classical population profile, because it occurred in a 27-year-old non-East Asian male - an atypical demographic for ApHCM - and was initially misattributed to ischemic disease despite normal coronary angiography. Cardiac magnetic resonance imaging (MRI) ultimately proved essential in confirming the diagnosis and guiding management, underscoring the need to consider ApHCM even in young, non-Asian patients with chest pain and abnormal ECG findings. Misdiagnosis and unawareness of this entity can lead to unnecessary invasive investigations and delays in the appropriate management.

## Case presentation

A 27-year-old university student presented to the emergency department (ED) with an episode of sudden, sharp chest pain lasting approximately one hour. The pain radiated to his left arm but was not associated with dyspnea, palpitations, or diaphoresis. By the time he arrived at the hospital, his symptoms had completely resolved. He reported experiencing brief, self-limited episodes of similar chest pain over the preceding few months. He denied any history of smoking, recreational drug use, or family history of cardiovascular disease.

**Figure 1:**
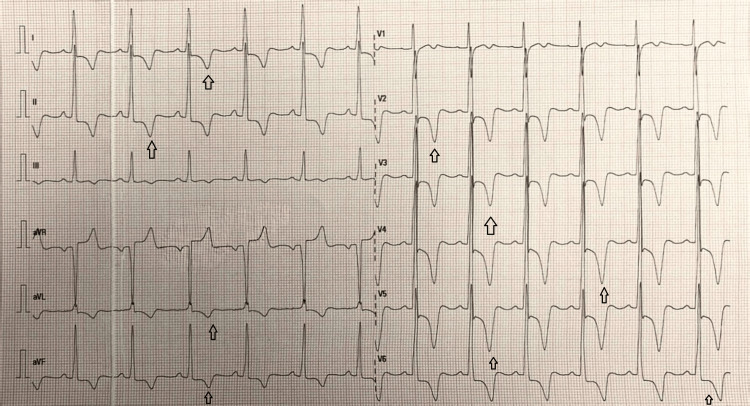
Giant, deeply inverted T-waves in the lead V2-V6, lead I, augmented vector left (aVL), and lead II, III, augmented vector foot (aVF) (black arrows)

**Figure 2:**
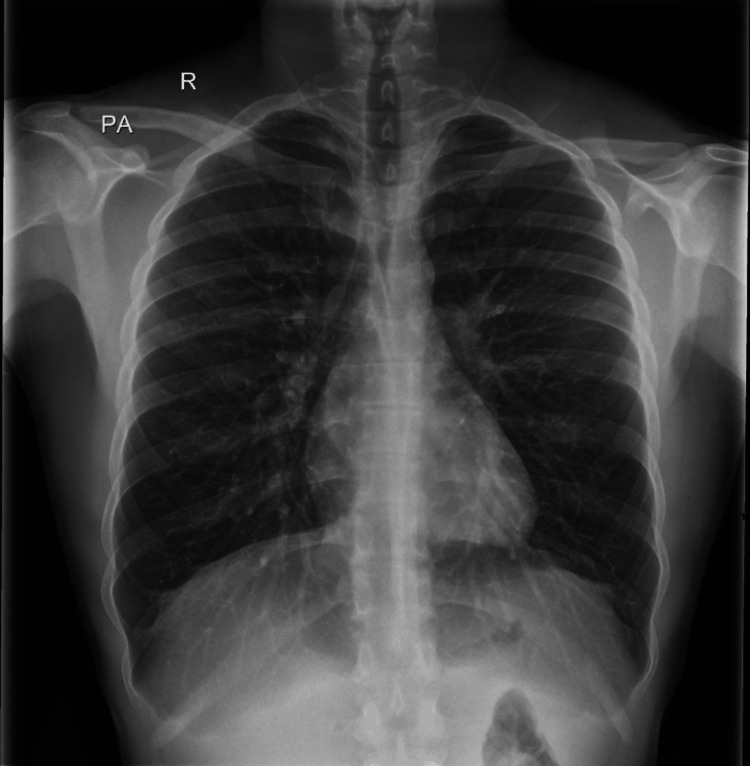
Normal chest X-ray

On examination, his vital signs were stable, and cardiovascular, respiratory, and systemic examinations were unremarkable. An ECG performed in the ED revealed high voltage features of tall R waves in lead aVF and precordial (V2-V6) leads as well as deep S waves in aVR, consistent with left ventricular hypertrophy. These changes were accompanied by giant, deeply inverted T-waves in the precordial (V2-V6), as well as in lateral (I, aVL) and inferior (II, III, aVF) leads, with the cardiac axis remaining within the normal range (Figure 1). Initial laboratory workup, including serial troponins and a complete metabolic panel, was within normal limits (Table 1). A chest radiograph showed no abnormalities (Figure 2). Despite normal cardiac enzymes, these ECG findings raised suspicion for non-ST elevation myocardial infarction (NSTEMI), and the cardiology team was consulted.

**Table 1:** Laboratory investigations ALT: alanine transaminase; AST: aspartate transaminase; MCV: mean corpuscular volume; WBC: white blood cell

Test	Result	Normal Range
WBC count	8 ×10^9^/L	4-10 ×10^9^/L
Hemoglobin	12.0 g/dL	13-17 g/dL
Platelets	340 ×10^9^/L	150-450 ×10^9^/L
MCV	90 fL	83-101 fL
Serum sodium	136 mmol/L	135-145 mmol/L
Serum potassium	4.1 mmol/L	3.5-5.0 mmol/L
Chloride	101 mmol/L	98-107 mmol/L
Bicarbonate	25 mmol/L	22-28 mmol/L
Urea	5.1 mmol/L	2.5-7.8 mmol/L
Creatinine	60 μmol/L	45-105 μmol/L
AST	41 U/L	15-50 U/L
ALT	45 U/L	5-55 U/L
Troponin I (First)	0.01 µg/L	<0.04 µg/L
Troponin I (Second)	0.01 µg/L	<0.04 µg/L
Troponin I (Third)	0.01 µg/L	<0.04 µg/L

**Figure 3:**
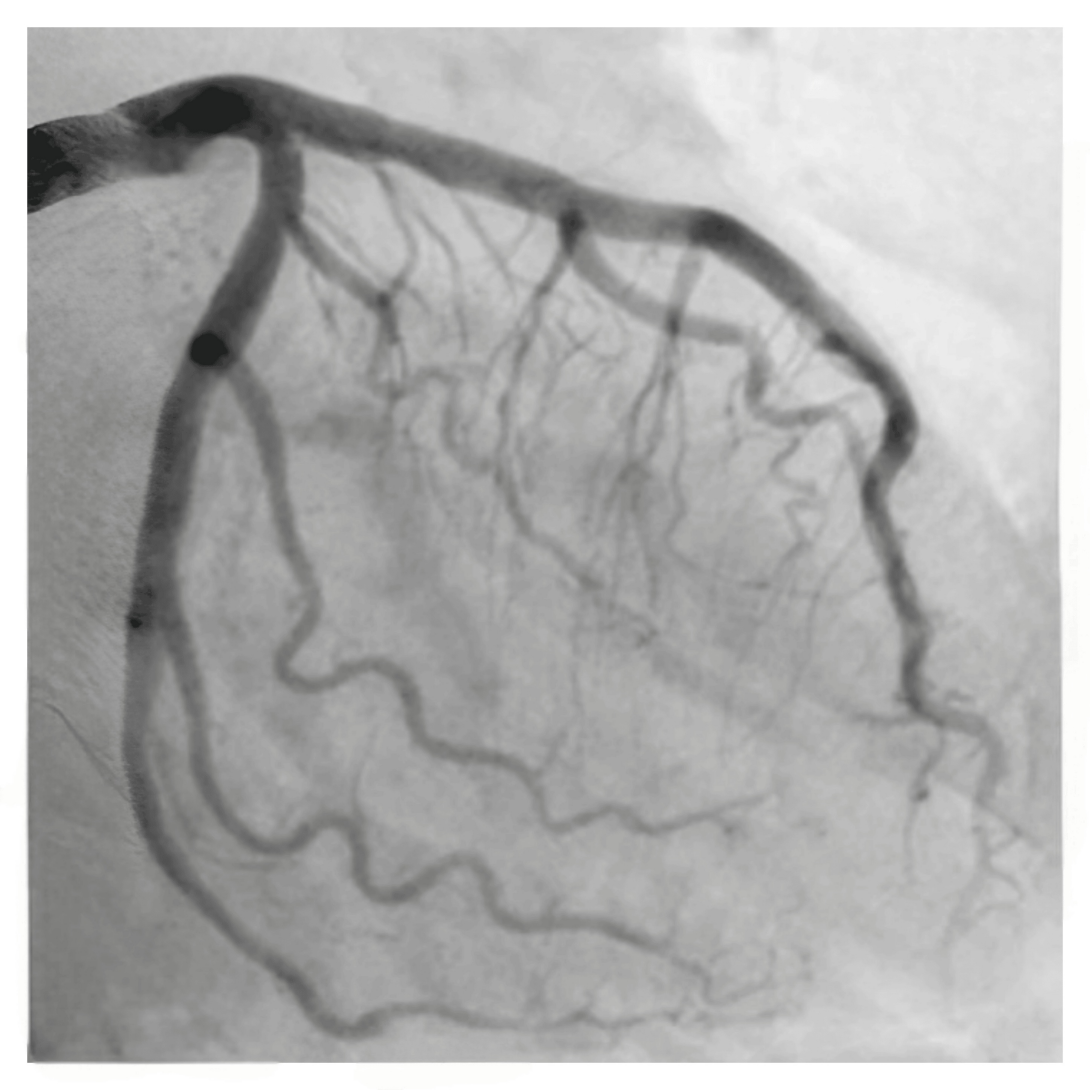
Normal coronary angiogram

**Figure 4:**
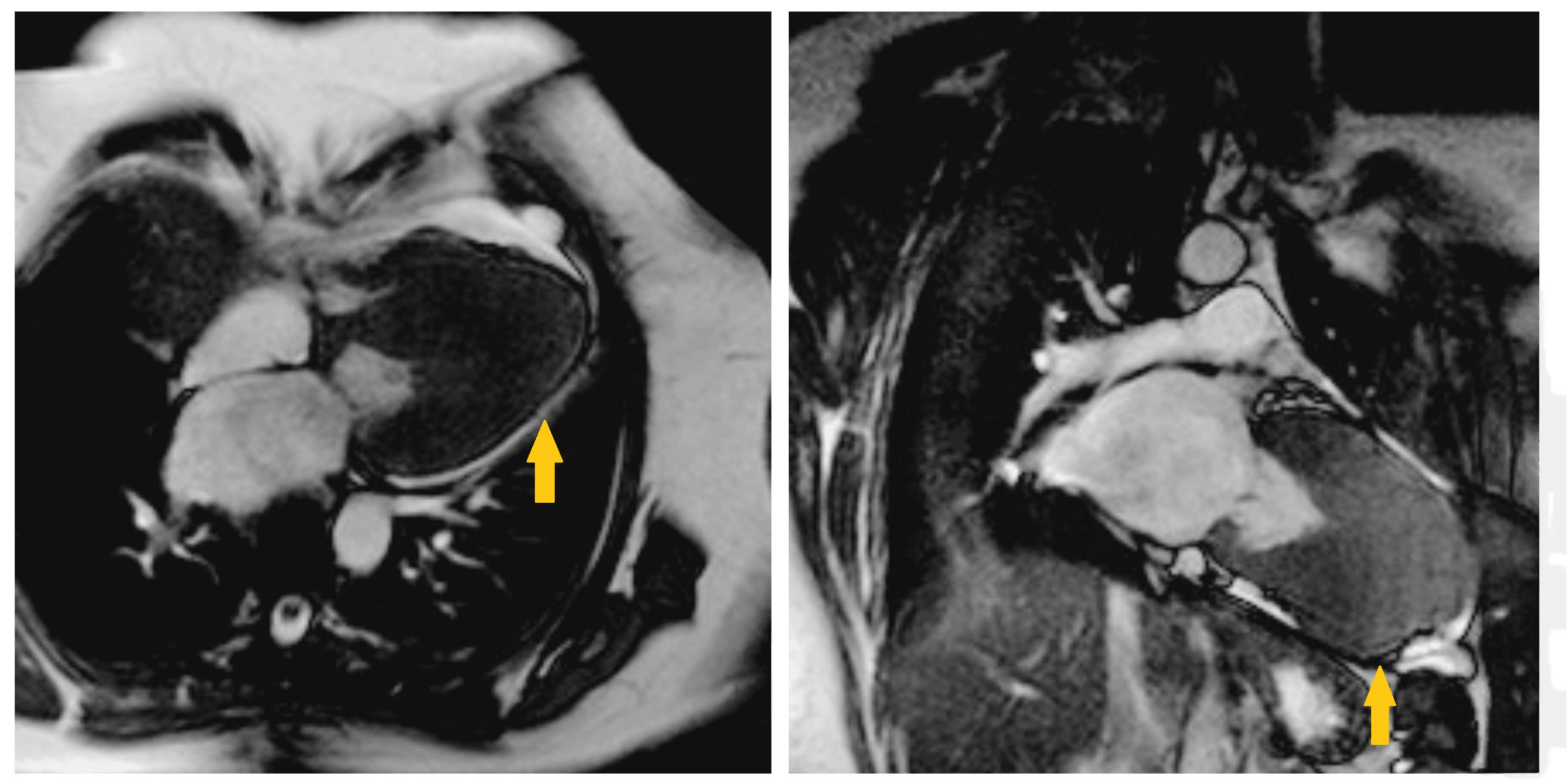
Cardiac MRI showing apical and mid-segment left ventricular hypertrophy (yellow arrows)

A transthoracic echocardiogram revealed normal left ventricular systolic function with an ejection fraction of 55%, and notable asymmetric thickening of the left ventricular apex without evidence of left ventricular outflow tract (LVOT) obstruction. To rule out obstructive coronary artery disease, the patient underwent coronary angiography, which demonstrated normal coronary arteries (Figure 3). Given the persistent concern for cardiomyopathy based on echocardiographic and ECG findings, cardiac MRI was performed. The MRI confirmed the presence of left ventricular apical hypertrophy with preserved systolic function, consistent with ApHCM (Figure 4).

The patient was started on bisoprolol 2.5 mg daily for symptomatic control and to reduce myocardial oxygen demand. He was discharged with plans for regular follow-up in a cardiomyopathy clinic and lifestyle counseling.

## Discussion

ApHCM, also referred to as Yamaguchi syndrome, is a less common subtype of HCM in which the thickening of the heart muscle is predominantly confined to the left ventricular apex. Sakamoto was the first to report the condition's electrocardiographic pattern and echocardiographic results in Japanese patients in 1976, while Yamaguchi was the first to characterize the syndrome and its ventriculographic features in 1979 [1]. Although it was initially reported in Japan and remains more prevalent in East Asian populations, cases of ApHCM have been reported globally. It accounts for a small percentage of all HCM cases, generally between 3% and 10%. A recent large Western registry from the Cleveland Clinic reported 6.8% of their 6,785-patient HCM cohort had ApHCM, underscoring its relevance beyond East Asia [2]. Historical cross-country data likewise showed ApHCM in ~3% of US HCM patients compared with ~15% in Japanese cohorts, highlighting the ethnic/geographic gradient but confirming non-Asian prevalence [3].

Symptoms of ApHCM range from exertional chest discomfort and palpitations to shortness of breath and fainting episodes, though many individuals remain asymptomatic [1]. Chest pain, the most frequent complaint, may closely mimic angina due to increased muscle mass, impaired perfusion in the apex, or elevated filling pressures, rather than obstructive coronary artery disease. This resemblance often leads to misdiagnosis, particularly in young patients who lack traditional cardiovascular risk factors [4,5].

ECG is often the first clue in raising suspicion for ApHCM. A distinctive finding is the presence of large, deeply inverted T-waves in the precordial leads, often with increased R-wave amplitude [6]. These changes are sometimes misinterpreted as ischemic, especially in patients presenting with chest pain. However, when cardiac enzymes are normal and no obstructive lesions are seen on angiography, alternative diagnoses such as ApHCM must be considered (Table 2). In our case, the ECG findings and normal coronary angiography helped expand the differential diagnosis early in the evaluation [6].

**Table 2:** Key differentiating features between apical hypertrophic cardiomyopathy and myocardial ischemia aVF: augmented vector foot; LAD: left anterior descending artery; LCx: left circumflex artery; LV: left ventricle; MRI: magnetic resonance imaging; RCA: right coronary artery Table credits: Abdul Rehman

Feature	Apical Hypertrophic Cardiomyopathy	Myocardial Ischemia
T-wave inversion pattern	Marked, symmetric, often very deep (“giant”) inversions, most prominent across the mid-to-lateral chest leads (V3-V6) and inferior (II, III, aVF) leads	T-wave inversions follow the distribution of the affected coronary artery (e.g., anterior leads in LAD disease, inferior leads in RCA/LCx disease)
Q waves	May show small, non-infarct “pseudo-Q waves” in chest leads	True pathologic Q waves appear in the infarcted regions
ST-segment changes	Usually minimal or absent; occasionally mild depression	Dynamic ST elevation or depression, depending on acute or chronic ischemia
Cardiac troponins	Typically remain within the normal range (unless there is superimposed ischemia)	Elevated with a characteristic rise-and-fall in acute infarction
Coronary angiography	Coronary arteries are usually unobstructed	Affected vessels often show significant stenosis or occlusion
Cardiac imaging (echocardiography/MRI/angiography)	Hypertrophy localized to the LV apex; “spade-shaped” cavity configuration on ventriculography or MRI	Wall motion defects localized to the territory supplied by the diseased vessel

Transthoracic echocardiography (TTE) typically serves as the initial imaging modality in ApHCM. It often reveals thickening confined to the left ventricular apex and may show a characteristic "spade-like" shape of the cavity during end-diastole. However, echocardiographic limitations - especially in visualizing the apex clearly - can lead to inconclusive results. In such cases, cardiac MRI provides high-resolution visualization of the ventricular morphology and is now considered a more definitive imaging modality for ApHCM [7,8]. In our case, MRI confirmed the diagnosis by demonstrating localized hypertrophy at the apex and excluding other structural abnormalities such as an aneurysm.

In patients with ApHCM, coronary CT angiography (CCTA) can be considered as a valuable, non-invasive option for evaluating coronary artery disease. CCTA provides detailed anatomical insight into myocardial and coronary morphology, effectively visualizing intramural coronary arteries and myocardial bridges, which are common in ApHCM [9]. Additionally, CCTA has demonstrated high diagnostic accuracy compared to invasive coronary angiography in detecting significant coronary stenoses, and its use is associated with reduced rates of major adverse cardiovascular events, overall mortality, and procedural complications in patients with stable disease. In contrast, although invasive coronary angiography remains the definitive modality for identifying and treating obstructive coronary lesions and is indispensable when intervention is being considered, it is invasive and carries a greater procedural risk and limited assessment of myocardial structure [10].

Treatment of ApHCM focuses on symptom management and risk reduction. Medications, such as beta-blockers or calcium channel blockers, are typically prescribed to improve diastolic relaxation and reduce myocardial oxygen consumption [11]. In patients with high-risk features, such as a history of syncope, family history of sudden cardiac death, or documented arrhythmias, implantable cardioverter defibrillators (ICDs) may be indicated [11].

ApHCM often has a more favorable prognosis than other forms of HCM, but patients may still develop atrial or ventricular arrhythmias, impaired diastolic function, apical aneurysm, and, in rare cases, sudden cardiac death. Ongoing follow-up should include Holter monitoring, stress testing, and repeat imaging to track the disease course. Because of its genetic nature, screening first-degree relatives with ECG and echocardiography is also recommended. Structured surveillance supports early detection of complications and guides individualized management. Predictors of poor outcomes include early age at diagnosis, positive family history, and the presence of heart failure symptoms beyond NYHA Class I [12].

Our case emphasizes the value of ECG interpretation in early diagnosis of ApHCM. Presence of atypical but characteristic ECG patterns, such as deep T-wave inversions and high-voltage R waves, should raise suspicion for ApHCM, especially in younger individuals without any risk factors for coronary artery disease. Cardiac MRI plays a key role in the definitive diagnosis of ApHCM, especially when echocardiographic findings are inconclusive, offering precise characterization of myocardial structure and helping guide the appropriate management and risk assessment.

Ultimately, timely identification of ApHCM through thoughtful evaluation of ECG and strategic use of cardiac MRI can lead to accurate diagnosis, reduce unnecessary procedures, and prevent potential long-term complications.

## Conclusions

Our case underscores the importance of considering ApHCM in the differential diagnosis of chest pain in young patients with abnormal ECG findings, even when conventional markers such as troponins are normal. Standard clinical pathways typically direct such patients toward invasive coronary angiography to exclude obstructive coronary artery disease, an approach rooted in the higher prevalence of ischemic heart disease and the relative rarity of HCM. However, this case illustrates how reliance on these algorithms can delay the recognition of ApHCM, particularly in atypical patients outside the classic demographic and presentation profile.

Early cardiac MRI should be considered when young patients present with chest pain and striking ECG abnormalities, such as giant T-wave inversions, despite normal troponins. Timely MRI not only prevents unnecessary invasive procedures but also enables accurate diagnosis and tailored management of this rare but clinically significant condition.
